# Retinal oximetry measures systemic hypoxia in central nervous system vessels in chronic obstructive pulmonary disease

**DOI:** 10.1371/journal.pone.0174026

**Published:** 2017-03-22

**Authors:** Thorunn Scheving Eliasdottir, David Bragason, Sveinn Hakon Hardarson, Charles Vacchiano, Thorarinn Gislason, Jona Valgerdur Kristjansdottir, Gudrun Kristjansdottir, Einar Stefánsson

**Affiliations:** 1 University of Iceland, Reykjavik, Iceland; 2 Department of Ophthalmology, The National University Hospital of Iceland, Reykjavik, Iceland; 3 Department of Anesthesiology, Landspítali—The National University Hospital of Iceland, Reykjavik, Iceland; 4 School of Nursing, Duke University, Durham, North Carolina, United States of America; 5 Department of Respiratory Medicine and Sleep (E7), Landspítali–The National University Hospital of Iceland, Reykjavík, Iceland; Charité - Universitätsmedizin Berlin, GERMANY

## Abstract

**Background:**

Determination of the blood oxyhemoglobin saturation in the retinal vessels of the eye can be achieved through spectrophotometric retinal oximetry which provides access to the state of oxyhemoglobin saturation in the central nervous system circulation. The purpose of this study was to test the capability of the Oxymap T1 oximeter to detect systemic hypoxemia and the effect of supplemental oxygen on retinal vessel oxyhemoglobin saturation.

**Methods:**

Oxygen saturation of hemoglobin in retinal arterioles and venules was measured in 11 subjects with severe chronic obstructive pulmonary disease (COPD) on long term oxygen therapy. Measurements were made with and without their daily supplemental oxygen. Eleven healthy age and gender matched subjects were measured during ambient air breathing for comparison of oxyhemoglobin saturation in retinal arterioles and venules. Retinal arteriolar oxyhemoglobin saturation in COPD subjects inspiring ambient air was compared with finger pulse oximetry and blood samples from radial artery.

**Results:**

COPD subjects had significantly lower oxyhemoglobin saturation during ambient air breathing than healthy controls in both retinal arterioles (87.2%±4.9% vs. 93.4%±4.3%, p = 0.02; n = 11) and venules (45.0%±10.3% vs. 55.2%±5.5%, p = 0.01). Administration of their prescribed supplemental oxygen increased oxyhemoglobin saturation in retinal arterioles (87.2%±4.9% to 89.5%±6.0%, p = 0.02) but not in venules (45.0%±10.3% to 46.7%±12.8%, p = 0.3). Retinal oximetry values were slightly lower than radial artery blood values (mean percentage points difference = -5.0±5.4, 95% CI: -15.68 to 5.67) and finger pulse oximetry values (-3.1±5.5, 95% CI: -14.05 to 7.84).

**Conclusions:**

The noninvasive Oxymap T1 retinal oximetry detects hypoxemia in central nervous system vessels in patients with severe COPD compared with healthy controls. The instrument is sensitive to changes in oxygen breathing but displays slightly lower measures than finger pulse oximetry or radial artery measures. With further technological improvement, retinal oximetry may offer noninvasive “on-line” measurement of oxygen levels in central circulation in general anesthesia and critically ill patients.

## Introduction

The eye is a window to systemic and central nervous system circulation. Its transparent anatomical structure provides a unique opportunity for direct noninvasive observation of arterial blood oxyhemoglobin saturation of retinal vessels; part of the central nervous system circulation. The retina has two separate vascular systems which differ anatomically and physiologically: the retinal circulation which supplies the inner retina and the choroidal circulation which supplies the avascular outer layers of retina. Both these circulations derive from the ophthalmic artery which is the first branch of the internal carotid artery[1] on its way carrying oxygen rich blood from the aorta to the brain. The central retinal artery derives from the ophthalmic artery and runs centrally within the optic nerve before it divides into four major arterioles, each supplying one quadrant of the inner retinal tissue.[2] Retinal arterioles are embryologically, anatomically and physiologically similar to cerebral arterioles[3,4] but branches of the central retinal artery lack innervation.[1] In the inner layers of the retina, vessel blood flow autoregulation is governed by myogenic and metabolic mechanisms[5] and similar to the cerebral vascular response[1] reacts within seconds to a reduction in perfusion pressure.[6] In the presence of shock[7] and severe hypoxia[8], cerebral and ocular[1,9] perfusion is preserved by peripheral vasoconstriction and redistribution of blood flow from lower priority organs to the central nervous system.[7] As a result of this redistribution of blood flow, measurement of oxyhemoglobin saturation by peripheral pulse oximetry may be unreliable [10] whereas measurement in retinal vessels may be a more direct indicator of cerebral oxyhemoglobin saturation.

In a variety of clinical settings including the operating room, intensive care unit, emergency department, motor vehicle accidents or combat injury, patients may suffer cardiovascular or other forms of shock. Measurement of oxyhemoglobin saturation by retinal oximetry may be a more reliable and relevant method to determine vital organ oxygenation and guide resuscitation in these shock patients than are measurements of oxyhemoglobin saturation in the peripheral circulation.

Dual wavelength spectrophotometric noninvasive retinal oximetry allows direct measurement of inner retinal vessel oxyhemoglobin saturation. It has shown good repeatability and sensitivity [11,12] and been widely used for retinal imaging and evaluation of retinal vessel oxyhemoglobin saturation in healthy subjects[11] and patients with several eye diseases.[13,14]

Moreover, retinal oximetry has shown sensitivity to changes in oxyhemoglobin saturation in patients with systemic hypoxemia. Traustason et al. measured hypoxia in retinal arterioles and venules in people with chronic systemic hypoxemia secondary to Eisenmenger syndrome [15]. The retinal measures correlated with both femoral artery oxyhemoglobin saturation, and finger oxyhemoglobin saturation. Recently, Palkovits and associates reported hypoxia of retinal vessels in people with severe chronic obstructive pulmonary disease COPD. [16]According to their findings, the retinal oxyhemoglobin saturation correlated with both capillary earlobe blood gas sample and finger pulse oximeter measurements.

The goal of the current study was to determine the value of retinal oximetry for detecting central and systemic hypoxemia. We compare retinal vessel oxyhemoglobin saturation in patients with severe COPD with healthy subjects. In addition, we evaluated the ability of the retinal oximetry device to measure changes in retinal vessel oxyhemoglobin saturation during variable oxygen breathing. Finally we compare retinal oximetry to radial artery blood sample and finger pulse oximetry values in the COPD group. This is the first report comparing retinal oximetry with direct arterial blood gas analysis in hypoxic COPD patients.

## Methods

### Design

**Fig 1:**
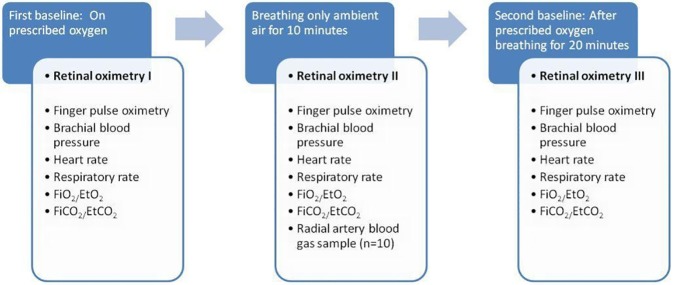
Order of experimental conditions.

The study was approved by the National Bioethics Committee of Iceland (06-084-V7) and the Icelandic Data Protection Authority (2012010136AT/-) and adhered to the tenets of the Declaration of Helsinki. Oxyhemoglobin saturation in retinal arterioles and venules in subjects with severe COPD were compared to healthy controls while breathing ambient air. In addition, the COPD subjects were exposed to 3 successive inspired gas conditions: 1) prescribed supplemental oxygen; 2) ambient air and; 3) prescribed supplemental oxygen again. Under each condition, blood pressure, finger pulse oximetry, heart rate, respiratory rate, fraction of inspired oxygen (FiO_2_), end-tidal carbon dioxide (EtCO_2_) and retinal oximetry images were obtained. Radial arterial blood samples for blood gas analysis were drawn during the ambient air breathing period only (Fig 1).

### Procedures

Patients with severe COPD [GOLD (Global Initiative in Obstructive Lung Disease) stage 3 or 4] as classified by a forced expiratory volume of less than 50% of predicted in one second (FEV_1_ < 50%) were enrolled in the study after signing a written informed consent. All COPD subjects were on long term oxygen therapy and had portable concentrator devices that supplied their prescribed oxygen supplementation (Luxfer, Salford M50 3XE, UK). Patients are prescribed long term oxygen therapy based on meeting the international criteria for experiencing sustained hypoxia;[17] generally defined as an arterial oxyhemoglobin saturation of < 90%. Study subjects’ medical histories were obtained from their patient records. All COPD subjects were in a stable condition, received their ambulatory pulmonary care at the National University Hospital in Iceland and had a baseline finger pulse oximetry of greater than 89% on their prescribed supplemental oxygen therapy. All COPD subjects were judged clinically to have sufficient respiratory reserve capacity to tolerate withholding their supplemental oxygen for a period of 15 minutes. Study exclusion criteria included: anticoagulation therapy with measured coagulation factors outside the normal range; signs or symptoms of a coronary arterial disease; history of congestive heart failure, atrial fibrillation, carotid stenosis, stroke, brain tumor, mental illness, diabetes mellitus or any eye disease. All COPD participants underwent a comprehensive eye examination before participating in the study.

Subjects were seated comfortably in a chair while breathing their prescribed supplemental oxygen from a portable concentrator device. A finger pulse oximeter sensor was applied to a finger for which a stable reading could be acquired and an initial peripheral arterial oxyhemoglobin saturation value and heart rate were obtained (Datex- Ohmeda OxyTip+ Healthcare, Finland). The patient’s portable oxygen concentrator device and nasal cannula were then replaced with a dual nasal cannula system (Flexicare Medical Limited, UK) connected to an oxygen cylinder with the flow meter set at the subjects prescribed flow rate. The cannula was also connected to a respiratory gas analyzer (Datex-Ohmeda D-LCC15.03, Planar Systems Inc., Beaverton Oregon, USA) with the ability to continuously monitor the subjects respiratory rate and measure the fraction of inspired oxygen (FiO_2_) and end-tidal carbon dioxide (EtCO_2_). Finger pulse oximetry, heart rate, respiratory rate, FiO_2_ and EtCO_2_ were continuously measured throughout the study. A non-invasive blood pressure cuff was applied over the brachial artery and blood pressure measurements were obtained at three different study time points (Omron M6 Comfort [HEM-7000-E]; Omron Healthcare Europe, Hoofddorp, The Netherlands). Systolic and diastolic blood pressure values were used to calculate mean arterial pressure (MAP) using the formula: (1/3 systolic blood pressure) + (2/3 diastolic blood pressure). After obtaining the first set of vital signs, and the initial finger pulse oximetry, FiO_2_ and EtCO_2_ (Table 1), the subjects’ pupils were dilated with 1% tropicamide (Mydriacyl, S.A. Alcon-Couvreur N.V., Puurs, Belgium).

**Table 1:** Basic physiological parameters (mean ± SD) at first baseline (on prescribed oxygen prior to the first retinal oximetry image), then after 10 minutes of only ambient air breathing and at second baseline after a recovery period of 20 minutes with oxygen breathing.

	Oxygen therapy	Ambient air breathing	Oxygen therapy
	First baseline		Second baseline
Physiological parameters	n = 10^1^	n = 11	n = 11
SBP (mmHg)	133 ± 21	127 **±** 19	129 **±** 13
DBP (mmHg)	82 **±** 15	78 **±** 10	82 **±** 11
MAP (mmHg)	99 **±** 15	94 **±** 11	97 **±** 11
SpO2 (%)	94 **±** 4	90 **±** 3	95 **±** 2
Heart rate (bpm)	82 **±** 10	77 **±** 13	76 **±** 12
RR (min-1)	18 **±** 4	15 **±** 3	15 **±** 3
	n = 9^2^	n = 10	n = 10
FiO2 (%)	39 **±** 20	21 **±** 3	43 **±** 15
EtCO2 (%)	34 **±** 8	33 **±** 7	33 **±** 8

SBP, systolic blood pressure; DBP, diastolic blood pressure; MAP, mean arterial blood pressure; SpO2, finger pulse oximetry; RR, Respiratory rate; FiO2, concentration of inhaled oxygen; EtCO2, end-tidal carbon dioxide.

n = 10^1^,One participant was not breathing the prescribed supplemental oxygen on arrival. Therefore no basic physiologal parameters at first baseline from that subject are presented in the table.

n = 9^2^,Reliable measures of FiO_2_ and EtCO_2_ from one of the participants were not possible to acquire due to the subject being a mouth breather.

Following pupil dilation, baseline retinal oximetry images were obtained using the Oxymap T1 retinal oximeter with subjects inspiring their prescribed supplemental oxygen therapy (first baseline period). The supplemental oxygen was then discontinued and the subject inspired ambient air at (close to) sea level for 10 minutes (ambient air period) followed by acquisition of the second set of retinal oximetry images. The prescribed supplemental oxygen was then re-instituted for a period of 20 minutes (second baseline period) and the final set of retinal oximetry images were obtained (Fig 1).

### Retinal oximetry

**Fig 2:**
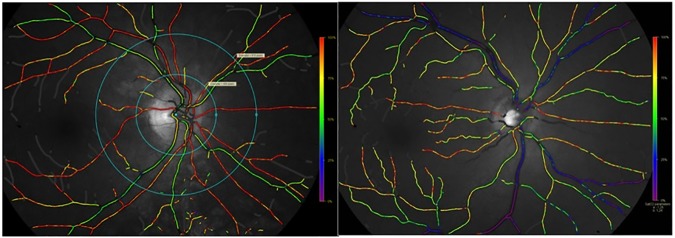
Pseudocolour oximetry images of a retina. A. Pseducolour image of a retina with the optic disc centered in a healthy subject. Vessel segments between the two circles are selected for analysis. The colours indicate oxyhemoglobin saturation as seen on the scale on the right. In general, arterioles are orange to red, indicating oxyhemoglobin saturation of about 90–100%. Venules may vary from blue to yellow but are normally green, indicating oxyhemoglobin saturation of approximately 50–60%. B. Retinal vessel oxyhemoglobin saturation in a patient with severe COPD after 10 minutes of ambient air breathing. The oximetry image indicates hypoxia of retinal arterioles and venules.

Determination of blood oxyhemoglobin saturation in retinal vessels is based on the fundamental principle that oxyhemoglobin and deoxyhemoglobin have different light absorption spectra. In the retinal oximetry application of this principle, an image of the fundus is obtained with the Oxymap T1 oximeter (Topcon TRC-50DX; Topcon Corporation, Tokyo, Japan) which has been described in detail elsewhere. [11,12] It is based on a conventional fundus camera (Topcon TRC-50DX; Topcon Corporation, Tokyo, Japan) connected to a custom-made optical adapter. A beam splitter is coupled with two digital cameras (Diagnostic Instruments Inc.) that capture a 50° view of the fundus. The retinal oximeter automatically acquires two monochrome images at different wavelengths; one at an isosbestic wavelength (570 nm) that is insensitive to oxyhemoglobin saturation and the other at oxyhemoglobin sensitive wavelength (600 nm). Specialized software (Oxymap Analyzer 2.4, version 6813) is then used to automatically select vessel points for measurement of light intensity inside and outside the vessel for calculation of optical density and optical density ratio. Optical density ratio has a nearly linear inverse relationship to oxyhemoglobin saturation [18–20] and thus enables calculation of blood oxyhemoglobin saturation. The system was calibrated by setting the mean oxyhemoglobin saturation in the retinal arteries of young healthy individuals at 92.2% and the mean for retinal venules at 57.9%.[11,21] The Oxymap analyzer software quantifies the oxyhemoglobin saturation and presents it in pseudo-color on a fundus image (Fig 2). Image acquisition and oximetry analysis were performed according to a standard protocol.[11] In brief, the same experienced researcher captured all the retinal images in a dark room with subjects sitting in front of the fundus camera. Five images of the right eye were taken first and then of the left eye at each of the three time periods (first baseline, ambient air breathing and second baseline). The average time for obtaining images of each eye was approximately 30 seconds. The first good quality image with the optic disc centered in the image was selected for analysis. Retinal arterioles and venules of the right eye with a minimum of 465 micrometers (μm) in length (50 pixels; one pixel is approximately 9.3 μm) were selected for calculation of mean oxyhemoglobin saturation. The vessel segments were chosen within an area that was demarcated by the two blue circles. The circles have diameters equal to 1.5 disc diameter and 3 disc diameter. Vessels that measured below approximately 75 μm in diameter (8.0 pixels) were excluded from calculation of the mean oxyhemoglobin values due to the fact that smaller vessels produce more variability and measurement artifacts. [22] Identical image acquisition and oximetric analyses were performed for the healthy subjects.

### Radial artery blood gas analysis

A modified Allen´s test[23] was performed on the non-dominant hand to confirm arterial competency. Following the second retinal vessel imaging session during which ambient air was inspired for 10 minutes, an arterial blood sample was drawn (BD Preset with needle, Becton, Dickinson and Company, UK) and sent for immediate blood gas analysis using co-oximetric blood gas analysis (ABL 800,Radiometer A/S, Husum, Denmark).

### Statistical analysis

Data analysis was performed using the Prism version 5 (GraphPad Software Inc, LaJolla, California, USA). A power analysis indicated that 7 subjects were required to detect a difference of 3 percentage points (%) in oxyhemoglobin saturation between retinal oximetry measurements of subjects inspiring ambient air versus supplemental oxygen with a power of 90%. Two-tailed paired *t-*tests and repeated measures one–way ANOVA were used for comparison of means. Dunnett's and Tukey´s multiple comparison post tests were carried out to compare pairs of group means. A p value of < 0.05 was considered to be significant. The data are presented as mean ± SD (95% confidence interval (CI)). Bland-Altman plots were used to determine the levels of agreement between the different measurement modalities, i.e. retinal oximetry, arterial blood samples and pulse oximetry. The degree of error, defined as the difference between the measurement values, was assessed in terms of bias and variability, where bias was calculated as the average difference between the measurements and variability was calculated as the mean bias ± 1.96 standard deviations.

## Results

Eleven (11) Caucasian subjects (7 female, 4 male) with severe COPD were enrolled in the study. The mean age was 70.4 ± 5.4 years and the range was 66 to 82 years. The control group data consisted of 11 age and gender matched healthy subjects (age 69.6 ± 4.9 years, range 64 to 80 years) selected from a group of 120 patients who had undergone retinal vessel imaging and oxyhemoglobin saturation determination while breathing ambient air prior to the current investigation. Of the 11 COPD subjects, one was not breathing the prescribed supplemental oxygen on arrival and therefore there was no first baseline measurement for this subject and we were unable to obtain an arterial blood gas sample from another. Therefore, data from 10 subjects was included in the statistical analysis at each study time period for comparison of mean and standard deviation within the COPD group. In addition, one COPD subject was a mouth breather and therefore we were unable to acquire sufficient FiO_2_ and EtCO_2_ data from the dual nasal cannula at any of the three study periods. All 11 COPD subjects were included in the ambient air breathing comparisons with the healthy controls.

### COPD subjects compared to healthy controls

**Fig 3:**
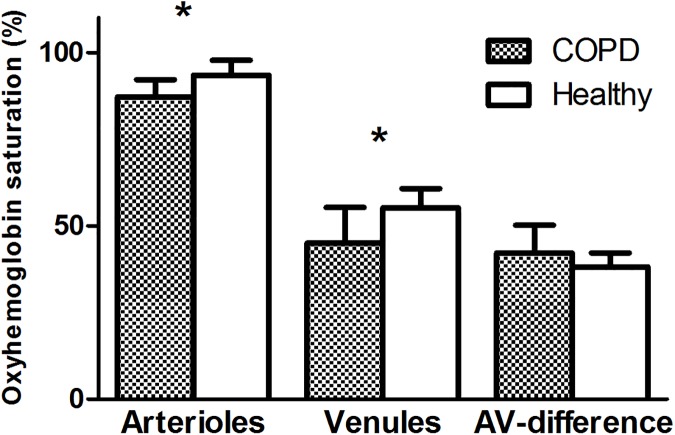
Comparison of arteriole, venule and arteriole-venule (A-V) oxyhemoglobin saturation difference between the total group of COPD subjects and healthy controls breathing ambient air. All values are presented as mean ± standard deviation. *Significant difference between COPD patients and healthy controls.

COPD subjects breathing ambient air (n = 11) had significantly lower mean oxyhemoglobin saturation compared to healthy controls (Fig 3) in both retinal arterioles (87.2±4.9% versus control = 93.4 ±4.3%, 95% CI:-11.31 to -1.02, p = 0.02, paired t test) and venules (45.0±10.3% versus control = 55.2±5.5%, 95% CI:- 17.95 to -2.37, p = 0.01). However, the arterial-venous oxyhemoglobin saturation difference was not significantly different (42.2±8.0% versus control = 38.2±4.0%, 95% CI: -1.98 to 9.98, p = 0.17).

Administration of prescribed supplemental oxygen significantly increased the oxyhemoglobin saturation in retinal arterioles (87.2%±4.9% to 89.5%±6.0%, 95% CI:-4.13 to -0.31, p = 0.02) but not in venules (45.0%±10.3% to 46.7%±12.8%, 95% CI: -5.15 to 1.76, p = 0.3). There were no differences between COPD and healthy subjects in diameter of arterioles (106.6±10.6 μm vs.114.2±10.9 μm, 95% CI: -17.06μm to 2.00μm, p = 0.11) or venules (147.7±14.1 μm vs. 153.4±15.1 μm, 95% CI: -24.28 μm to 12.85μm, p = 0.51) respectively.

### COPD subjects exposed to the experimental protocol

Table 2 summarizes the oxyhemoglobin saturation measurements in 10 COPD subjects from retinal vessel oximetry and finger pulse oximetry at the three study time periods and from radial artery co-oximetry during the ambient air breathing period. After cessation of supplemental oxygen breathing and 10 minutes of inspiring ambient air, the oxyhemoglobin saturation in retinal arterioles and finger oximetry values decreased significantly. Breathing of supplemental oxygen for 20 minutes (second baseline period) returned the retinal arteriole and finger oxyhemoglobin saturation values to near the first baseline values. Neither retinal venule oxyhemoglobin saturation, arterial-venous oxyhemoglobin saturation difference, nor retinal vessel diameter were significantly affected by the cessation or reapplication of supplemental oxygen breathing. There was no significant difference between first and second baseline in any of the oximetric measurements.

**Table 2:** Comparison of percent (%) oxyhemoglobin saturation between retinal vessels, finger pulse oximetry and radial artery blood with and without supplemental oxygen therapy in 10 patients with severe COPD. Arteriole and venule oxyhemoglobin saturation difference and diameters are also shown.

n = 10	Oxygen therapy	Ambient air for 10 minutes	Oxygen therapy	
	First baseline		Second baseline	
Retinal arterioles	91.0 **±** 4.5	87.5 **±** 5.1	90.0 **±** 5.9	* 1.03 to 6.05^a^ *-5.09 to -0.07^b^
Finger pulse oximetry	93.7 ± 3.6	90.6 **±** 2.8	94.7 **±** 2.5	* 0.14 to 6.05^a^ *-7.05 to -1.14^b^
Radial artery	__	92.5 **±** 3.6	__	
Retinal venules	47.6 **±** 12.7	45.6 **±** 10.6	46.5 **±** 13.5	ns -2.13 to 6.11^a^ ns -4.96 to 3.28^b^
AV- difference	43.4 **±** 10.6	41.8 **±** 8.3	43.5 **±** 9.6	ns -2.68 to 5.79^a^ ns -5.98 to 2.49^b^
Arteriolar diameter	107.8 **±** 18.4	104.7 **±** 8.7	102.6 **±** 10.1	ns -4.26 to 10.63^a^ ns -5.42 to 9.47^b^
Venular diameter	142.7 **±** 19.2	147.3 **±** 14.8	143.5 **±** 17.5	ns -13.10 to 3.85^a^ ns -4.66 to 12.29^b^

^**a**^ Oxygen therapy at first baseline versus ambient air breathing for 10 minutes

^**b**^ Ambient air breathing versus oxygen therapy at second baseline.

Values are mean ± SD. Repeated measures one-way ANOVA and Tukey´s multiple comparison post test. (CI: 95% Confidence interval of the difference).

* p < 0.05; ns, non-significant.

**Fig 4:**
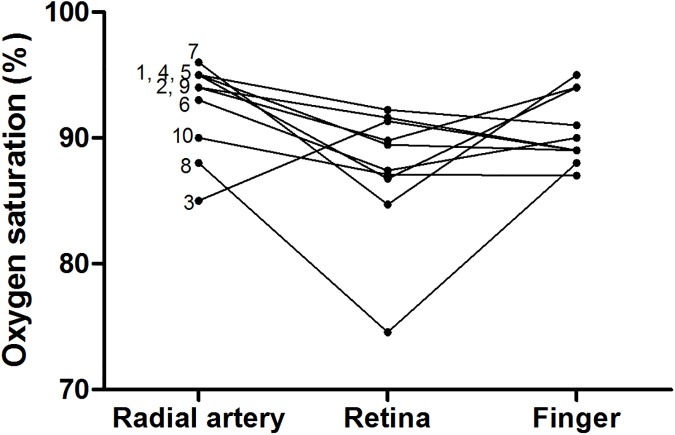
Comparison of 10 COPD subjects’ retinal arteriole, radial artery blood, and finger pulse oximetry oxyhemoglobin saturation after 10 minutes of ambient air breathing. Each number indicates a COPD subject with reference to Table 2. Each data point represents mean oxyhemoglobin saturation (%) in a single COPD subject. Repeated measures one-way ANOVA and Dunnett´s multiple comparison post test.

Data from 10 COPD subjects after 10 minutes of ambient air breathing (Fig 4) revealed that the mean oxyhemoglobin saturation in retinal arterioles was 87.5±5.1% compared to 92.5±3.6% in the radial artery (95% CI: -8.65 to -1.35, p<0.05) and 90.6±2.8% in the finger (95% CI: -6.75 to 0.54, p >0.05).

**Fig 5:**
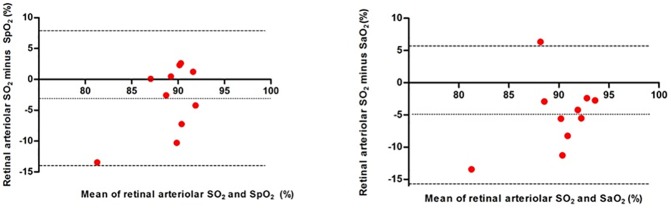
**Bland Altman plots comparing retinal arteriole oxyhemoglobin saturation values with A. finger pulse oximetry and B. radial artery blood during ambient air breathing in 10 patients with systemic hypoxemia due to severe COPD.** SO2, retinal arteriole oxyhemoglobin saturation; SpO2, finger pulse oximetry; SaO2, radial arterial oxyhemoglobin saturation. Dotted lines indicate mean difference between measurements and dash lines indicate 95% limits of agreement.

Bland Altman plots were constructed to compare retinal arteriolar oximetric oxyhemoglobin saturation values with finger pulse oximetry and radial artery blood. Retinal arteriole oxyhemoglobin saturation during ambient air breathing revealed a bias and limit of agreement of -3.1±5.5; 95% CI: -14.05 to 7.84 as compared with finger pulse oximeter measurements and -5.0±5.4; 95% CI: -15.68 to 5.67 as compared with the radial artery blood sample valus (Fig 5A and 5B). During ambient air breathing, retinal arteriolar saturation was not significantly correlated (S1 Fig) with either radial artery saturation (p = 0.45) or finger oximetry (p = 0.76).The COPD subjects as a group had a mean partial pressure of oxygen (PaO_2_) and carbon dioxide (PaCO_2_) of 61.3±10.5mm Hg and 42.2%±5.9 mm Hg respectively, a mean bicarbonate of 26.4 ±3.4 mEq/L, and a mean pH of 7.4±0.0.

## Discussion

Noninvasive spectrophotometric retinal oximetry was able to detect reduced oxyhemoglobin saturation in patients with severe COPD breathing ambient air compared to healthy subjects. The oximeter also captured an increase in retinal arteriole oxyhemoglobin saturation when the COPD subjects inspired supplemental oxygen. Our findings of increased oxyhemoglobin saturation in retinal arterioles and unchanged AV-difference during supplemental oxygen breathing are in agreement with the study of Palkovits and associates[16] on patients suffering from severe COPD in a stable condition. Supplemental oxygen therapy improves global oxygen delivery and therefore the oxyhemoglobin saturation of retinal arterioles as demonstrated in our COPD subjects. A high FiO_2_ amplifies the oxygen flux from choriocapillaries, not only to the outer retina but to the inner retina as well [24] which may have contributed to the improved arteriolar oxyhemoglobin saturation in the inner retinal tissue with supplemental oxygen breathing.

Our results demonstrate that while there are some intra-individual differences, in general retinal arteriole oxyhemoglobin saturation values were lower than those measured with finger oximetry and from radial artery blood samples across the three study conditions. The Bland Altman plots demonstrated this tendency of retinal oximetry to produce lower oxyhemoglobin saturation but manifested a fair agreement with the other two modalities. The difference could simply be due to calibration. If the difference is real, however, one possible explanation for lower retinal arterial saturation is that the relatively small retinal arterioles that are measured, simply lose more oxygen through their walls by diffusion than arteries measured in the finger or arm (the retinal measurements are made on segments of vessels that stretch for some length into the retina as shown on Fig 2). Additionally, the central retinal artery and vein lie side by side within the optic nerve and counter current exchange of oxygen may take place between the arteriole and venule and this could result in slightly lower saturation in retinal arterioles compared with large arteries. Aside from retinal oximetry in systemic hypoxic disease, a significant correlation has been shown between retinal vessel oxyhemoglobin saturation and finger pulse oximetry following induced hypoxemia in healthy subjects.[25] The sensitivity of retinal oximetry has been validated in pigs exposed to acute hypoxemia that showed good correlation with both femoral artery oxyhemoglobin saturation and intra-vitreal oxygen tension.[26]

Retinal oximetry has previously been tested over a wide range of oxyhemoglobin saturation levels[15,24] and validated against arterial blood gas analysis[15,26] and intra-vitreal partial pressure of oxygen.[26] The validation process has in general revealed good correlation and an approximately linear relationship between the measured parameters. The Oxymap T1 estimates retinal oxyhemoglobin saturation based on calibration factors, which assume that mean oxyhemoglobin saturation in healthy subjects is 92.2% for arterioles and 57.9% for venules. These reference values were originally obtained with a laboratory oximeter, which was calibrated in vitro and are somewhat lower than quoted normal values in the systemic circulation. [27] Inhalation of 100% oxygen by facemask (FiO_2_ of approximatley 96%) in healthy subjects has shown to increase the oxyhemoglobin saturation in retinal arterioles to 94.5%±3.8 and 76.2%±8.0% in venules[24] which is closer to normal mixed venous oxyhemoglobin saturation during ambient air breathing. Sustained hypoxemia secondary to Eisenmenger syndrome is reflected by subnormal oxyhemoglobin saturation in retinal arterioles of 81% ± 9% and 44% ± 12% in venules.[15] Despite a number of experimental spectrophotometric measurements of arteriolar and venous retinal oxyhemoglobin saturation in human subjects, no “normal” gold standard values exist due to the invasive nature of the process needed to obtain the parameters necessary to determine such normal values *in vivo*. This barrier is also responsible for the lack of a definitive threshold for retinal vessel hypoxemia. Nevertheless, retinal oximeter measurements can provide valuable information with respect to relative and trend oxyhemoglobin saturation values in retinal arterioles and venules.

The reason for the differences in oxyhemoglobin saturation of retinal arterioles and peripheral vascular beds need to be elucidated. Reevaluation of the instrument calibration and further studies on larger numbers of healthy subjects and patients suffering from systemic hypoxic condition are needed to address the issues of absolute arterial oxyhemoglobin saturation and retinal arteriolar relative values.

Retinal oximetry quantifies oxygen delivery[15,16] and oxygen consumption in the central circulation and is a promising technique for patient monitoring in the acute care clinical setting in the future. One of the most significant obstacles to the use of this technology in the clinical setting is the sheer size and bulk of the oximeter apparatus however, work on miniaturization of the device has begun with a development of a hand held device that needs to be tested in real life clinical settings in the future.

### Conclusion

Noninvasive retinal oximetry detects lower oxyhemoglobin saturation in central nervous system vessels in patients with severe COPD compared with healthy controls. It is sensitive to changes in concentration of inspired oxygen but measured values are slightly below both arterial blood and finger pulse oximetry values. With further technological improvements and development of a hand held device, retinal oximetry may eventually offer noninvasive “real-time” measurement of oxyhemoglobin saturation levels in the central circulation in the anesthesia and critical care settings.

## Supporting information

S1 Fig Correlations of retinal oximetry with radial artery oxyhemoglobin saturation and finger pulse oximetry. (TIF)
