# Supplementary figures and images for: Retinal oximetry measures systemic hypoxia in central nervous system vessels in chronic obstructive pulmonary disease
Source: PLoS One. 2017 Mar 22;12(3):e0174026. doi: 10.1371/journal.pone.0174026 (PMC5362093; doi:10.1371/journal.pone.0174026)

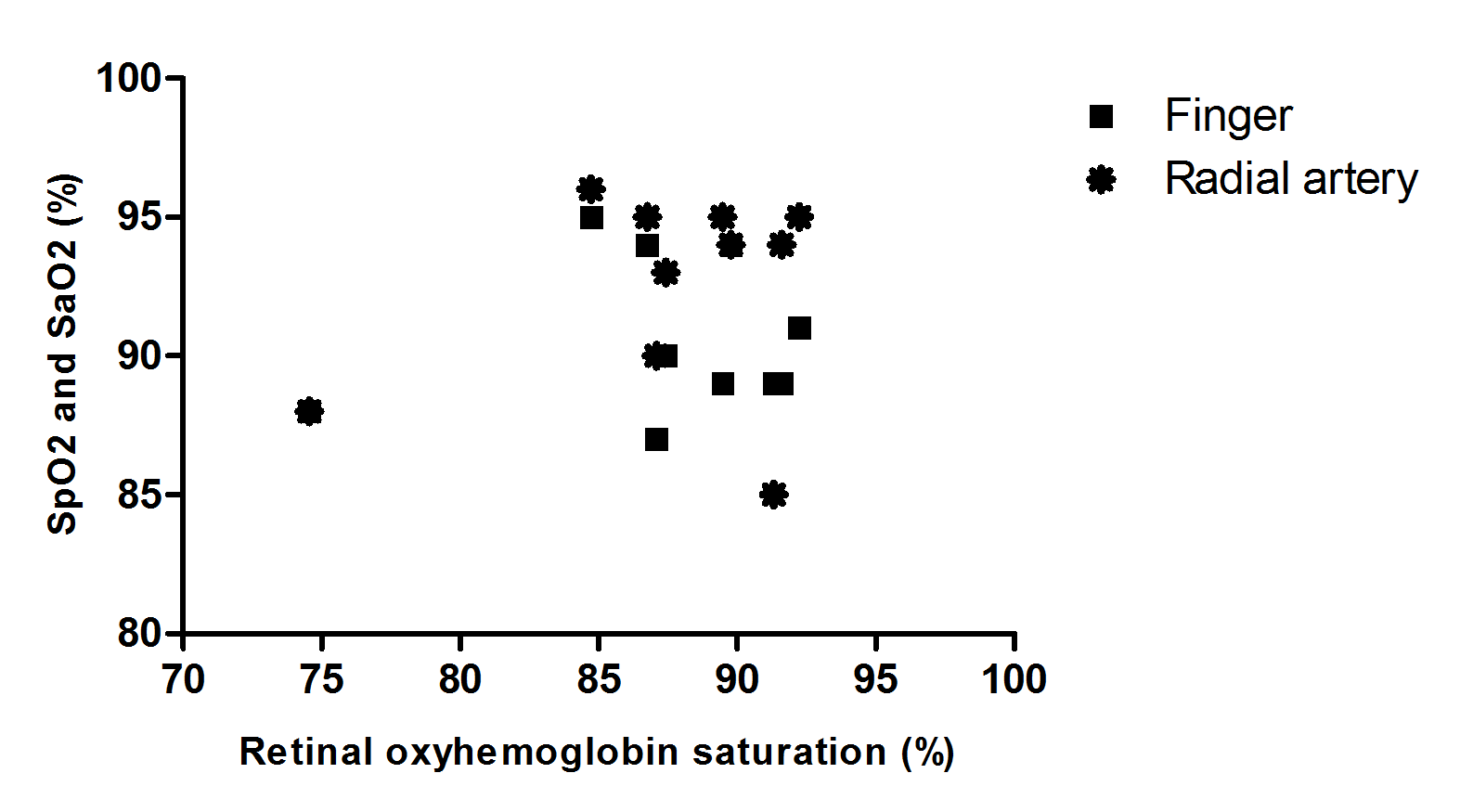

Supplement: S1 Fig — (TIF) [file pone.0174026.s001.tif]
